# The Pathogenic R3052W BRCA2 Variant Disrupts Homology-Directed Repair by Failing to Localize to the Nucleus

**DOI:** 10.3389/fgene.2022.884210

**Published:** 2022-05-30

**Authors:** Judit Jimenez-Sainz, Adam Krysztofiak, Jennifer Garbarino, Faye Rogers, Ryan B. Jensen

**Affiliations:** Department of Therapeutic Radiology, Yale University School of Medicine, New Haven, CT, United States

**Keywords:** BRCA2, R3052W, homology-directed repair, nuclear localization, DNA repair, DSS1, RAD51

## Abstract

The BRCA2 germline missense variant, R3052W, resides in the DNA binding domain and has been previously classified as a pathogenic allele. In this study, we sought to determine how R3052W alters the cellular functions of BRCA2 in the DNA damage response. The BRCA2 R3052W mutated protein exacerbates genome instability, is unable to rescue homology-directed repair, and fails to complement cell survival following exposure to PARP inhibitors and crosslinking drugs. Surprisingly, despite anticipated defects in DNA binding or RAD51-mediated DNA strand exchange, the BRCA2 R3052W protein mislocalizes to the cytoplasm precluding its ability to perform any DNA repair functions. Rather than acting as a simple loss-of-function mutation, R3052W behaves as a dominant negative allele, likely by sequestering RAD51 in the cytoplasm.

## Introduction

**FIGURE 1:**
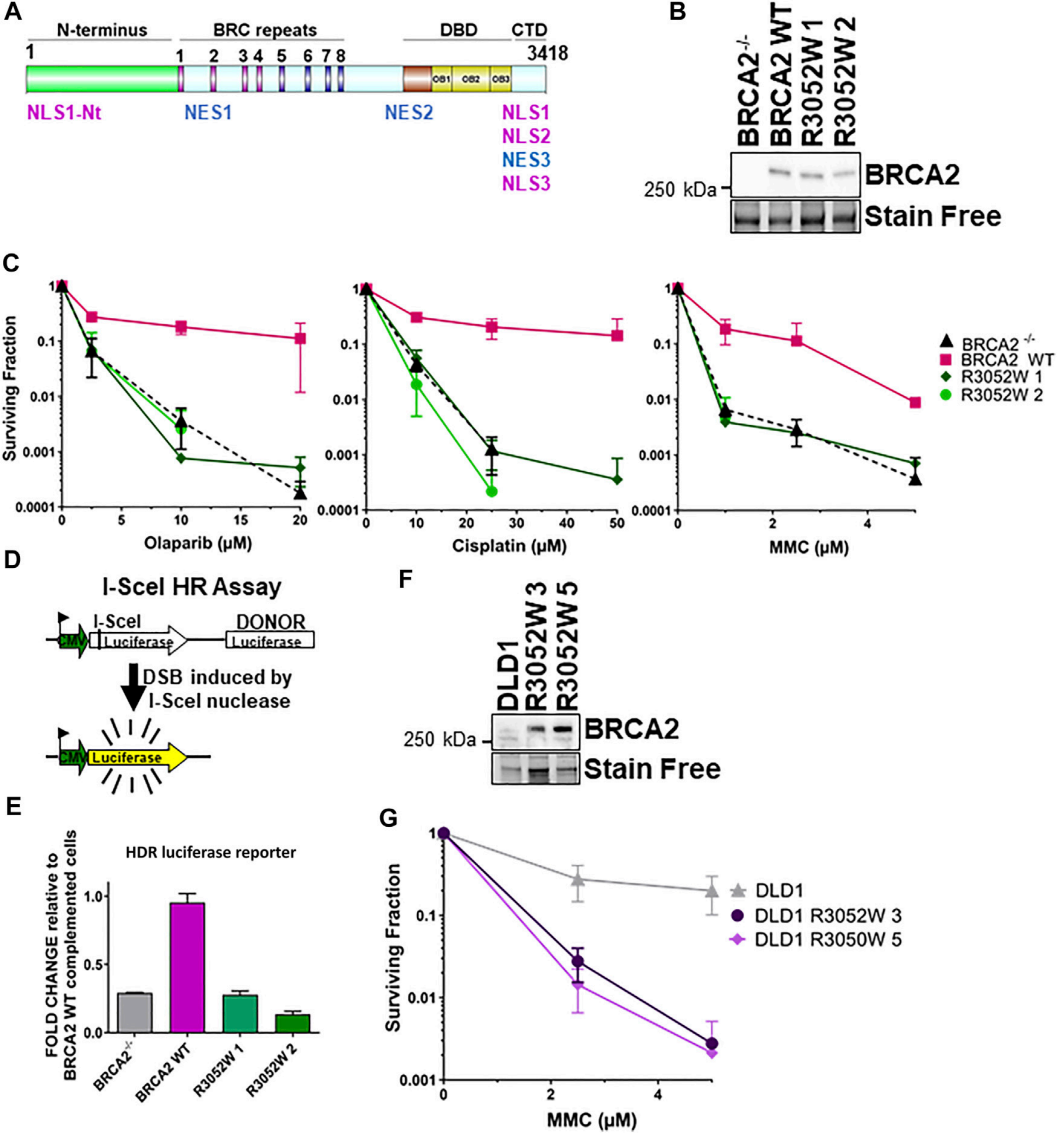
The BRCA2 R3052W mutation fails to complement chemotherapeutic sensitivity and homology-directed repair functions in BRCA2 knockout cells. Overexpression of R3052W in DLD1 parental BRCA2 wild-type cells confers sensitivity to MMC DNA damage. **(A)** BRCA2 protein schematic depicting domain organization: N-terminus, BRC repeats DNA binding domain (DBD), and C-terminal domain (CTD). BRCA2 nuclear localization and export sequences are listed. NLS1-Nt (433–436 aa), NLS1 (3,265–3,270 aa) NLS2 (3,311–3,317 aa), NLS3 (3,381–3,385 aa), NES1 (1,383–1,392 aa), NES2 (2,682–2,698 aa) and NES3 (3,270–3,280 aa). **(B)** Western blot of total cellular lysates from DLD-1 BRCA2^−/−^ cells stably transfected with full-length BRCA2 cDNA constructs: BRCA2 Wild Type (WT) and BRCA2 R3052W (1 and 2 correspond to two independent clones). BRCA2 cDNAs contain a 2XMBP tag on the N-terminus and were detected with an MBP antibody. 2XMBP-BRCA2 (470 kDa). Stain Free is a protein-loading control. **(C)** Clonogenic survival analyses of BRCA2 WT versus the two independent R3052W clones after treatment with Olaparib, cisplatin, and mitomycin C. **(D)** Schematic of I-SceI nuclease-induced DSB HDR luciferase assay. **(E)** Quantification of luciferase activity (normalized to BRCA2 WT as 1). Error bars are SD (*n* = 5). **(F)** Western blot of total cellular lysates from DLD-1 parental cells (these cells express a wild-type allele of BRCA2) stably transfected with R3052W (3 and 5 correspond to two independent clones) full-length 2XMBP-BRCA2 cDNA constructs. BRCA2 was detected with an MBP antibody. **(G)** Clonogenic survival analyses of DLD parental clones upon treatment with mitomycin C.

BReast CAncer Susceptibility Gene 2 (BRCA2), identified in the early 1990s, is a hereditary breast and ovarian cancer gene which codes for a 3,418 amino acid protein with several identifiable functional and structural domains and numerous interacting partners (Wooster et al., 1994; Wooster et al., 1995; Easton et al., 1997) (Figure 1A and reviewed in the work of Jimenez-Sainz and Jensen, 2021). The BRCA2 DNA binding domain (DBD) was crystallized in complex with DSS1 (PDB ID: 1IYJ, 736 amino acids) illuminating an alpha-helical domain, tower domain, and three tandem oligonucleotide/oligosaccharide-binding folds (OB-folds 1–3) (Yang et al., 2002). DSS1 is a small acidic protein (70 amino acids) proposed to drive nuclear localization of BRCA2 and promote BRCA2 protein stability (Gudmundsdottir et al., 2004; Kojic et al., 2005; Li et al., 2006). BRCA2, together with DSS1, facilitates the exchange of replication protein A (RPA) for RAD51 on resected single-stranded DNA (ssDNA) (Yang et al., 2002; Zhao et al., 2015). The crystal structure also revealed that BRCA2 can bind an ssDNA oligonucleotide making several contacts with the OB2 and OB3 folds located in the DBD (Yang et al., 2002). Our previous study demonstrated that a BRCA2 protein fragment encompassing the DBD and C-terminal domain (CTD) can localize to the nucleus, bind 3′tail DNA, and is capable of minimally stimulating RAD51-mediated DNA strand exchange (Chatterjee et al., 2016).

BRCA2 possesses several putative nuclear localization (NLS) and nuclear export (NES) sequences (Spain et al., 1999; Yano et al., 2000; Han et al., 2008; Jeyasekharan et al., 2013) distributed throughout the domains of the protein. Three of the NLS (NLS1, NLS2, and NLS3) located at the C-terminus of BRCA2 have previously been proposed as the primary NLSs for the nuclear localization of BRCA2 (Spain et al., 1999) (**see** Figure 1A). BRCA2 nuclear localization is essential for the protein to carry out homology-directed repair (HDR), replication fork protection, and cell cycle checkpoint functions (Scully and Livingston, 2000; Venkitaraman, 2002; Schlacher et al., 2011; Mijic et al., 2017; Taglialatela et al., 2017; Eckelmann et al., 2020; Rickman et al., 2020). Importantly, truncating mutations upstream of NLS1, 2, and 3 are predicted to lead to loss of nuclear localization and, thus, are associated with HDR dysfunction and cancer predisposition (Spain et al., 1999; Sakai et al., 2009).

The R3052 residue of BRCA2 is located in exon 23 situated between OB folds 2 and 3 in the DBD. The R3052W mutation (c.9154 C > T) co-segregates with BRCA2-related disease (breast, ovarian, and prostate cancer) in several families (Farrugia et al., 2008; Gomez Garcia et al., 2009) and has been classified as pathogenic according to the ClinVar and Breast Cancer Information Core (BIC) databases (Variation ID: 52763, rs45580035, ExAC 0.006%).

Prior functional studies implicated R3052W as pathogenic due to HDR impairment and the inability to rescue cell viability in a mouse embryonic stem cell model (Farrugia et al., 2008; Kuznetsov et al., 2008; Guidugli et al., 2013; Cunningham et al., 2014; Hendriks et al., 2014; Shimelis et al., 2017; Guidugli et al., 2018; Hart et al., 2019). In this study, we analyzed the functional effects of the R3052W missense mutation incorporated into our full-length BRCA2 construct and expressed the mutant protein in a viable BRCA2 deficient human cell model. The full-length BRCA2 R3052W protein appears stable as expression levels are similar to the WT protein. We find that R3052W is unable to rescue the sensitivity of BRCA2 deficient cells to crosslinking agents and PARP inhibitors, is defective in executing HDR, and these defects arise due to the mislocalization of the protein in the cytosol. To our surprise, expression of the R3052W mutant in a BRCA2 WT background exacerbates genomic instability and sensitivity to DNA damage suggesting a dominant negative effect rather than a simple loss-of-function mutation. We postulate that cytosolic localization of R3052W could be due to protein aggregation or nuclear import defects, but not to the loss of DSS1 binding leading to active nuclear export, as we confirm that DSS1 binding remains intact in the R3052W mutant.

## Materials and Methods

### Constructs

Point mutation R3052W was cloned into the phCMV1 2XMBP-BRCA2 and phCMV1 2XMBP-BRCA2 DBD + CTD only sequences, *via* site-directed mutagenesis (cloning strategy and primers available upon request). We verified the putative recombinant clones through restriction digestion and sequencing analysis. The previously described 2XMBP tag (Jensen et al., 2010) was placed in-frame at the N-terminus of all proteins separated by an Asparagine linker and the PreScission Protease cleavage sequence. To clone N-terminal fusions of GFP and mCherry to BRCA2 WT and R3052W, 2XMBP tag was removed from the constructs and PCR products of GFP and mCherry were digested with KpnI/NotI and inserted in phCMV1 BRCA2 and R3052W constructs. All the constructs were verified by sequencing analysis.

### Cell Culture Transient Transfections

All culture media were supplemented with 10% fetal bovine serum (FBS). HEK293T cells were cultured in DMEM (source Jensen et al., 2010); DLD1 cells were cultured in RPMI. Transient transfections were carried out with Turbofect (Thermo Scientific) (2 μg of DNA, 6-well plates) in HEK293T cells and with JetOptimus (Polyplus Transfection) in DLD1 cells following the manufacturer’s protocol. DLD1 BRCA2^−/−^ cells 50–60% confluent in 24-well plates (15000 cells per well) were transiently transfected with 0.5 μg of 2XMBP DBD + CTD BRCA2 WT or 2XMBP DBD + CTD R3052W construct with jetOPTIMUS reagent (Polyplus). Then, 48 h post-transfection cells were processed for immunofluorescence. Calcium Phosphate (25 μg of DNA, 15 cm^2^ plate, see BRCA2 purification section) was used on large scale in HEK293T cells (Jensen et al., 2010). All cell lines were tested regularly for mycoplasma (Mycoalert, Lonza) and confirmed through STR profiling.

### Generation of Stable Cell Lines

Human colorectal adenocarcinoma DLD-1 BRCA2^−/−^ and DLD1 parental cells [Horizon Discovery, originally generated by (Hucl et al., 2008)] were stably transfected with 2 μg of DNA using Lipofectamine 3000 (Invitrogen). After 48 h, the cells were trypsinized and diluted 1:2, 1:4, and 1:8 into 100 mm plates containing 1 mg/ml G418. Single-cell colonies were picked into 96-well plates and subsequently cultured into 24-well plates, 12-well plates, and 6-well plates. Positive clones were isolated, and protein expression was detected by western blot and immunofluorescence analyses.

### Western Blots and Amylose Pulldowns

Human embryonic kidney HEK293T cells 70% confluent in 6-well plates were transiently transfected with 2 μg of the phCMV1 mammalian expression vector containing a 2XMBP fusion to the full-length of BRCA2 using TurboFect reagent (Thermo Scientific) (Jensen et al., 2010). 1ug of HA-DSS1 was transfected into the cells as described before (Zhao et al., 2015). 0.5 μg of 2XMBP empty construct was transfected into these cells and an untransfected well was also seeded as a negative control. The cells were lysed 48 h after transfection in 200 μL of lysis buffer: 50 mm HEPES (pH 7.5), 250 mm NaCl, 5 mm EDTA, 1% Igepal CA-630, 3 mm MgCl_2_, 10 mm DTT and protease inhibitor cocktail (Roche). Cell extracts were batch bound to amylose resin (NEB) for 2 h to capture the 2XMBP tagged BRCA2 proteins. Total cellular lysate aliquots were taken before batch binding for control analysis. Total cellular lysates and amylose pulldown samples were run on a 4–15% gradient SDS-PAGE TGX stain-free gel (Bio-Rad 456-8086), which was subsequently transferred to an Immobilon-P membrane (Merck Millipore IPVH00010) in 1X Tris/glycine buffer (diluted from 10X Tris/glycine buffer, Bio-Rad 161-0771). The membrane was blocked in 5% milk in 1X TBS-T (diluted from 10X TBS-T: 0.1 M Tris base, 1.5 M NaCl, 0.5% Tween-20). Washes and antibody incubations were done with 1X TBS-T. Primary mouse antibodies against MBP (NEB E8032L, 1:5,000), RAD51 (Novus Biologicals 14b4, 1:1,000), and primary rabbit antibody against BRCA2 (Abcam, ab27976), GAPDH (Sta Cruz Biotechnology 0411m #sc-47724, 1:1,000) and HA (Cell Signaling C2974 mAB#3724, 1:1,000) were used for western blotting. Membranes were then incubated with secondary mouse and rabbit antibodies (HRP-conjugated, Santa Cruz Biotechnology sc-516102, and sc-2004, respectively). Protein expression was visualized by incubating with Clarity Western ECL substrate (Bio-Rad 170-5061) for 3 min and scanning with a ChemiDoc MP imaging system (Bio-Rad).

### Clonogenic Survival Assay

Stable cell clones generated from DLD-1 BRCA2^−/−^ cells were serially diluted and seeded into 6-well plates at concentrations of 100 and 500 cells per well in triplicate for plating efficiency. Simultaneously, cells were seeded for treatment in 6-well plates at 1,000 and 10,000 cells per well in triplicate per treatment dosage. 24 h after seeding, cells were treated with indicated doses of mitomycin C (MMC, 1.5 mm stock in water) cisplatin (100 mm stock in DMSO) for 1 h in serum-free media, and Olaparib (50 mm stock in DMSO) for 24 h. Following treatment, media was aspirated, and cells were washed with 1X PBS and re-fed with fresh media containing FBS. Cells were cultured for 14 days to allow colony formation, after which they were stained with crystal violet staining solution (0.25% crystal violet, 3.5% formaldehyde, 72% methanol). Colonies containing 50 or more cells were scored and surviving fractions were determined.

### Homology-Directed Repair Luciferase Assay

The HDR luciferase reporter gene (gWiz.Lux-5′-3′Luc) was constructed from the parental vector gWiz Luciferase (Genlantis) as previously described (Chatterjee et al., 2016). An I-SceI site was created in the luciferase ORF by ligating the following annealed oligonucleotides: 5′SCEILUC (5′-CGC TAG GGA TAA CAG GGT AAT-3′) and 3′SCEILUC (5′-CGA TTA CCC TGT TAT CCC TAG-3′) into a BstBI site. The 7 amino acid insertion at amino acid 56 of the luciferase ORF ablates luciferase activity. A second luciferase ORF was ligated into the XmnI site 700 bp downstream from the first luciferase ORF (see Figure 1D). The second luciferase ORF lacks a promoter but can be utilized as a donor in homology-directed repair of the first luciferase ORF upon generation of a double-strand break by expression of the I-SceI nuclease. The pSce-MJ mammalian I-SceI expression vector was a kind gift from Dr. Fen Xia.

To perform the assay, cells were seeded into 6-well plates at 2.5 × 10^5^ cells/well. 24 h later, cells were transfected with 200 ng of gWiz.Lux-5′-3′Luc vector and 1 µg of the I-SceI expression vector using Lipofectamine 3,000. gWiz.Lux-5′-3′Luc or gWiz.Lux vectors were transfected alone and used as negative and positive controls, respectively. Cells were harvested 48 h post-transfection in 200 µL of lysis buffer described above prior to luminometer analysis. Luminescence was measured using an integration time of 5 s with 40 µL of the lysate plus 100 µL of luciferin substrate (One-Glo luciferase assay, Promega). Luciferase values were measured as independent duplicates in each experiment. The raw data were normalized to protein levels and the values plotted were calculated by setting the full-length BRCA2 mean value to 100%. The data presented the average of three independent experiments.

### Laser Microirradiation

Sterile gridded glass coverslips (#1.5H D263 Schott glass cat. #10816; ibidi inc.) were coated with collagen (cat. #125-50, Millipore-Sigma) by incubation at 37°C for 1 h. Subsequently, exponentially growing DLD1^−/−^, WT BRCA2, and R3052W stable cells were plated into coverslips and incubated overnight. After overnight incubation cells were treated with 10 μM BrdU (19–160; EMD Millipore) and incubated for additional 48 h prior to irradiation. Shortly before the irradiation, coverslips with cells were washed with PBS and placed in a 50 mm glass bottom (#1.5 glass) dish (P50G-1.5-30-F, Mattek Corp.) in a low-absorption medium (RPMI; 31053; Thermo Fisher Scientific). A dish with cells was mounted on a Leica TCS SP8 X microscope system (Leica Microsystems) inside an incubator chamber at 37°C with 5% CO2 supplementation. For laser microirradiation, a Leica HC PL APO 40 ×/1.30 Oil CS2 objective with a zoom factor of 0.75 was used to establish the expected field of irradiation, which encompassed up to 200 cells in a single frame. 40 horizontal stripe masks (5 px wide) were placed in a 512 px × 512 px view field. The cells were irradiated 55 times with a 405 nm diode laser at 95% with FRAP booster with a pixel dwell time of 3.75 µs. One full frame irradiation lasted 1.985 s, with a total irradiation time of 109.175 s for 55 iterations (130 Hz bidirectional, frame rate 0.503/sec). A laser power exiting the objective was equal to 1.14 J/sec. Consequently, each pixel of the irradiation masks was exposed to 4.275 µJ per iteration resulting in a total energy of 235.125 µJ. Our experimental conditions induce ssDNA and dsDNA breaks. The cells were not synchronized so only 20% of the cellular population showed RAD51 recruitment to the micro-irradiated area. After irradiation, cells were fixed and permeabilized with 3% formaldehyde, 0.2% Triton X-100 and 8% sucrose for 15 min at RT and were subject to immunofluorescent staining protocol.

### Immunofluorescence Staining, Imaging, and Quantification

Stable cell clones generated from DLD-1 BRCA2^−/−^ cells were grown on coverslips at 10^5^ cells/well in a 24-well plate for 24 h. Cells were washed twice with 1X PBS, fixed in 1% paraformaldehyde-2% sucrose in 1X PBS for 15 min at room temperature, washed twice with 1X PBS, permeabilized with methanol for 30 min at −20°C, then washed two more times with 1X PBS, and finally incubated with 0.5% triton in PBS for 10 min. Samples were then blocked with 5% BSA in 1X PBS for 30 min at room temperature followed by subsequent incubation with primary antibodies against MBP (NEB E8032L, 1:200), gammaH2AX (Millipore, Ser 139, clone JBW301, 05-636, 1:100) and RAD51 (Proteintech 14961-1-AP, 1:100 or Abcam ab63801) in 5% BSA-0.05% TritonX-100 at 4°C overnight. The next day, cells were washed three times with 1X PBS and incubated with goat anti-rabbit and anti-mouse secondary antibodies conjugated to the fluorophores Alexa-488 and Alexa-546 (Thermo Fisher Scientific A11034 and A11003, respectively; 1:1,000). Coverslips were washed three times with 1X PBS, incubated with 30 nm DAPI for 5 min, and mounted on slides with FluorSave reagent (Calbiochem 345789). Immunofluorescence images were taken using a Keyence BZ-X800E All-in-One Fluorescent Microscope with a 40x or 60x objective lens. Cells were either untreated as control or irradiated at 12 Gy using an X-Rad 320 Biological Irradiator and cells were collected at 6- and 24-hours post-irradiation for immunofluorescence protocol. For subcellular localization analysis of BRCA2 (red), RAD51 (green in Figures 3A, 4A and red in Figure 5) and DSS1 proteins, cells with nuclei (N), nuclei/cytosol (N/C) and cytosol (C) distribution of the proteins were scored and divided by the total number of cells (DAPI, blue) to obtain a ratio between 0 (no signal) to 1 (positive signal) and the ratios were represented. For micronuclei formation analysis all the micronuclei, as well as the total number of cells, were counted with DAPI staining and the percentage of cells with micronuclei was represented. At least 300 cells (3-5 microscope images in three independent experiments) were counted. For gammaH2AX and RAD51 foci quantification at least 300 cells (3-5 microscope images in three independent experiments) were counted. In the case of RAD51 foci analysis, cells with more than 5 foci within the nuclei were considered positive and in the case of gammaH2AX, cells with more than 10 foci within the nuclei were considered positive. Graph Pad PRISM version 9.3.1 was used to generate all graphs.

For microirradiation immunofluorescence staining cells were incubated for 2 h at RT in a blocking buffer solution containing: 5% normal goat serum (10000C; Invitrogen), 8% Sucrose, and 0.2% Triton X-100 in PBS. Subsequently, cells were incubated overnight at 4°C with primary antibodies: gammaH2AX (Millipore, Ser 139, clone JBW301, 05-636, 1:100) and RAD51 (Abcam ab63801). Following three washing steps with PBS +0.5% Triton X-100, cells were stained with secondary goat antibodies anti-rabbit Alexa-488 and anti-mouse Alexa-546 as indicated above. Next, cell nuclei were stained with DAPI 2.5 μg/ml; 1816957; Thermo Scientific) for 15 min at RT. Samples were washed three times with PBS +0.5% Triton X-100 for 5 min and then rinsed one time with PBS before mounting with DAKO Fluorescence Mounting Medium (S3023; Dako—Agilent Technologies).

Subsequently, images were acquired with a Nikon Eclipse Ti fluorescence microscope with a CFI Plan Apochromat Lambda 60x/1.4 Oil, WD 0.13 mm objective (Nikon Corporation), a CSU-W1 confocal spinning disk unit (50 µM disk pattern, Yokogawa), an iXon Ultra 888 EMCCD camera (Andor Technology), MLC 400B laser unit (Agilent Technologies) and NIS Elements 4.30 software (Nikon Corporation). Images were taken with three-quarters of the maximum intensity without overexposure. The pictures were saved as 1024 pixels × 1024 pixels, 16-bit multi-channel. nd2 files with no further editing. For stripes data quantification. nd2 files were additionally exported to 16-bit OME-TIFF format. For quantification of fluorescent intensities within damaged areas and nuclei overall, the ImageJ-based tool Stripenator was used (Oeck et al., 2019). For RAD51 protein fluorescent intensities, damaged area/background intensity ratios were calculated for each cell to normalize the damage intensity values to their own background. Consequently, if the damaged and background area have similar intensities because of no change in protein localization to the damaged site, a value of 1 would be obtained versus higher values if the damage was more intensely stained than the background and values lower than 1 if damage area was less intensely stained than the background. For the automatic quantification of RAD51 staining in the stripes, all cells in the same field were included so the difference in the average of RAD51 recruitment is smaller than expected due to the mixture of ssDNA and dsDNA breaks and the lack of cell cycle synchronization. Additionally, mean fluorescent intensities (MFIs) of RAD51 inside and outside of the cell nuclei were calculated using the ImageJ-based tool Focinator (Oeck et al., 2017). These ratios were normalized and are presented with respect to the area of field of view (220.16 microns × 220.16 microns) of each evaluated picture since on each analyzed picture was the different number of cells.

### Nuclear Export Analysis

For nuclear export inhibitor experiments, stable cell clones generated from DLD-1 BRCA2^−/−^ cells were grown on coverslips at 1 × 10^5^ cells/well in a 24-well plate for 24 h. Then, cells were treated with 50 ug/ml of leptomycin B for different time points (0, 2, 4, 6 h) and processed as in the immunofluorescence imaging section. YFP-c-abl (gift from Dr. Anthony Koleske) and Rev 1.4 MP2K2 GFP (gift from Dr. Ane Olazabal) constructs were included as positive controls of nuclear retention upon Leptomycin B treatment (Henderson and Eleftheriou, 2000). In CRM1 silencing experiments, stable cell clones generated from DLD-1 BRCA2^−/−^ cells were grown at 5 × 10^5^ cells/well in a 6-well plate for 24 h. Then, 25 nM siRNA for CRM1 (ON-TARGETplus siRNA Dharmacon Set of four: 09, 10, 11, 12) were introduced into the cells with Dharmafect. After 24 h, cells were reseeded on coverslips 1 × 10^5^ cells/well in a 24-well plate and kept for 24 h. The next day, cells were processed as in the immunofluorescence imaging section above. Non-targeting control was used as a negative control. siRNA for GAPDH was used as a positive control.

## Results

### The BRCA2 R3052W Variant Does Not Rescue Chemotherapeutic Sensitivity or Homology-Directed Repair Deficiency

Several families in the BIC database with evidence of genetic linkage indicate that the BRCA2 R3052W variant is a pathogenic mutation with a high probability of future cancer risk (Farrugia et al., 2008;Gomez Garcia et al., 2009;Mohammadi et al., 2009;Capanu et al., 2011;Cunningham et al., 2014). The crystal structure of the carboxy terminus of BRCA2 (Yang et al., 2002) places the R3052 residue at a potentially critical interface between OB folds 2 and 3 (Kuznetsov et al., 2008) in the DBD. The R3052 residue (see Supplementary Figure S1 for sequence alignment) is conserved amongst several different species suggesting that deviations from this amino acid are not tolerated throughout evolution. To gain further insight into the mechanistic nature of this variant, we expressed an N-terminal 2XMBP tagged full-length BRCA2 construct incorporating the R3052W mutation in a human DLD1 BRCA2 knockout cell line to directly compare against our previously generated wild-type (WT) complemented cell line (Chatterjee et al., 2016). Single-cell-derived stable R3052W clones had comparable BRCA2 protein expression levels to the WT BRCA2 complemented cell line (Figure 1B). BRCA2 deficient cells are sensitive to crosslinking agents and PARPi (Davies et al., 2001;Bryant et al., 2005;Chatterjee et al., 2016). To determine if the R3052W mutation could rescue BRCA2 deficient cells similar to the WT protein, survival assays were performed (Figure 1C). Examination of two independently derived stable clones expressing the BRCA2 R3052W protein resulted in the same level of surviving cellular fraction as the BRCA2 knockout cells (empty vector) suggesting a complete loss-of-function in response to PARPi and crosslinking agents (Figure 1C). Similarly, the R3052W expressing cells were unable to repair a split luciferase reporter construct designed to measure HDR repair of a single I-Sce-induced DNA DSB (Scheme Figure 1D) (Figure 1E). Together, these results suggest that the R3052W mutation is incapable of providing the canonical HDR cellular functions of BRCA2. Studies in other DNA repair proteins (e.g., ATM) postulated that missense variants could exert dominant negative effects, however, it was unknown if BRCA2 missense variants previously described could elicit such an effect and increase cancer risk in the heterozygous state or in the ectopic expression state (Tavtigian et al., 2009). To test whether the R3052W mutation could impact functions of the WT BRCA2 protein, we expressed R3052W (Figure 1F) in DLD1 parental cells (containing one endogenous BRCA2 WT allele) and interrogated the cellular sensitivity to mitomycin C. As shown in Figure 1G, R3052W ectopic expression decreased the survival of DLD1 parental cells suggesting a dominant negative effect. Our results point toward a pathogenic component of the R3052W mutation that somehow disrupts the normal cellular functions of WT BRCA2, rather than a simple loss-of-function allele.

### R3052W Increases Genomic Instability and Decreases RAD51 Foci Formation Upon Irradiation

**FIGURE 2:**
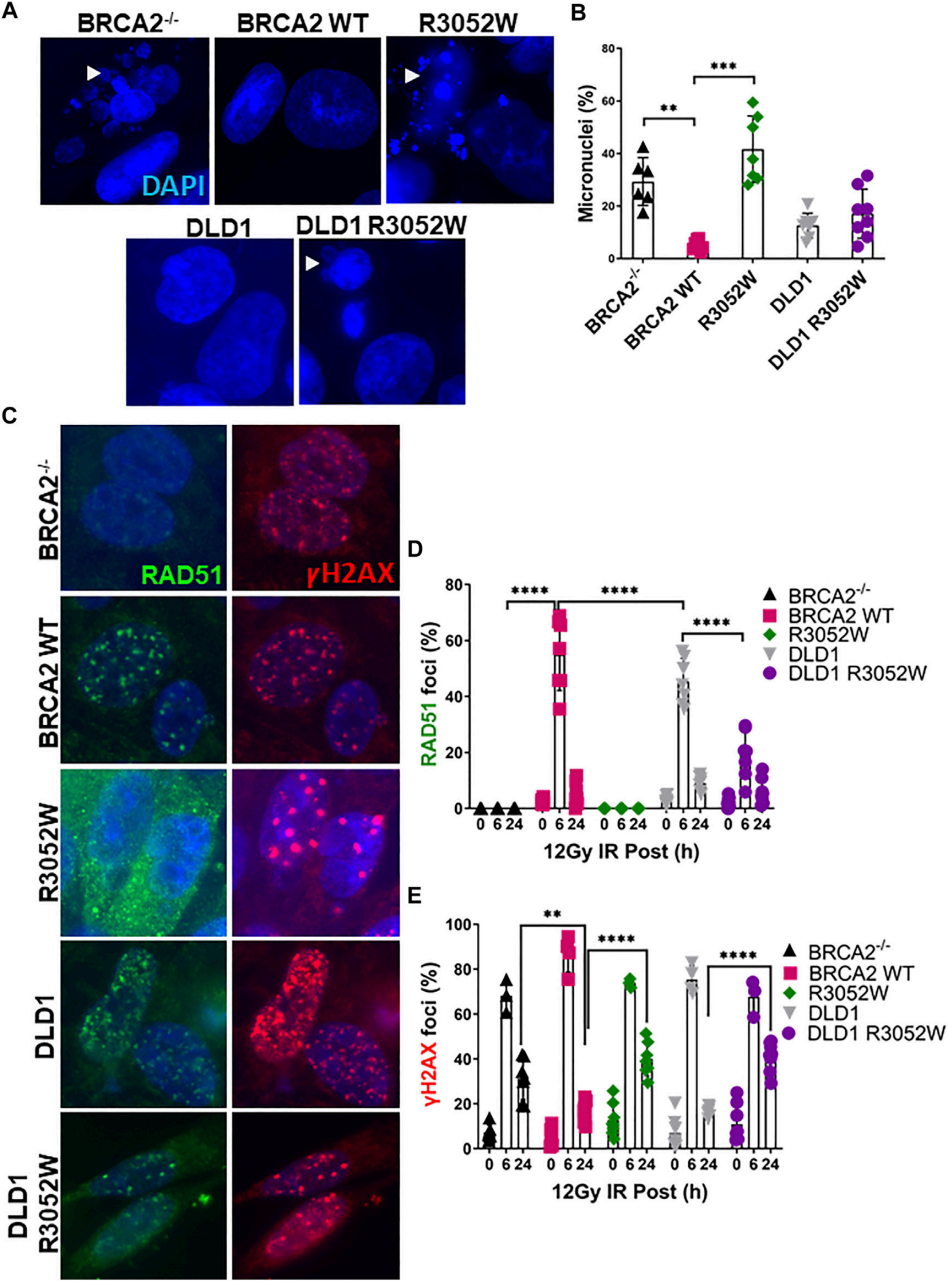
The BRCA2 R3052W mutation exacerbates micronuclei formation and interferes with RAD51 foci formation in DLD1 parental and BRCA2 wild-type cells. **(A)** Representative immunofluorescent images of micronuclei (blue) in a DLD1 BRCA2 knockout cell line (top) expressing WT or the R3052W mutant and parental DLD1 cells (bottom) compared to a stable cell line expressing the R3052W protein. **(B)** Quantification of the percentage of cells with micronuclei. **(C)** Representative immunofluorescent images of RAD51 (green) and gammaH2AX (red) foci, and DAPI staining to visualize nuclei (blue). Cells were fixed and imaged 6 h post-IR (12 Gy). **(D)** Quantification of the percentage of cells with RAD51 foci. **(E)** Quantification of the percentage of cells with gammaH2AX. (Quantification represents 3 independent experiments and statistical analysis t-test and one-way ANOVA. **p-value < 0.01, ***p value < 0.001, ****p value < 0.0001).

Previous studies in a conditional BRCA2 knockout mouse embryonic stem cell model and VC8 BRCA2 mutant hamster cells demonstrated that R3052W can not rescue cell viability or HDR activity (Farrugia et al., 2008; Kuznetsov et al., 2008). However, information regarding the role of R3052W in maintaining genomic integrity and HDR activity in human cells has been lacking. To address this gap, we irradiated human cells stably expressing either the WT or R3052W protein with 12 Gy to track micronuclei formation. Micronuclei contain chromosomes, or damaged chromosome fragments, not incorporated into the nucleus during cell division and have been previously used as a readout of genomic instability (Luzhna et al., 2013). Our results demonstrate that expression of R3052W increases the percentage of cells with micronuclei in both a BRCA2 deficient (BRCA2^−/−^) and a BRCA2 proficient background (DLD1) (Figures 2A,B). Next, we visualized RAD51 and gammaH2AX (γH2AX) foci formation as surrogates of HDR activation and DNA damage response respectively (Figure 2C) (Heddle et al., 1983; Ban et al., 2001; Haaf et al., 1995; Rothkamm et al., 2015; Chatterjee et al., 2016). RAD51 and γH2AX foci peaked at 6 h and were mostly resolved by 24 h following 12 Gy of ionizing radiation in WT complemented cells and the DLD1 parental cells (Figures 2C–E). However, R3052W expression in BRCA2 knockout cells displayed no visible nuclear RAD51 foci following ionizing radiation damage while γH2AX foci persisted at the 24 h timepoint. Interestingly, stable R3052W expression in DLD1 parental cells (with endogenous BRCA2) displayed a significantly lower percentage of cells with RAD51 foci at the 6 h time point and a higher proportion of cells with γH2AX foci at the 24 h timepoint unresolved, suggesting an HDR defect leading to a sustained DNA damage response **(** Figures 2C–E **).**

### R3052W Mislocalizes to the Cytosol

**FIGURE 3:**
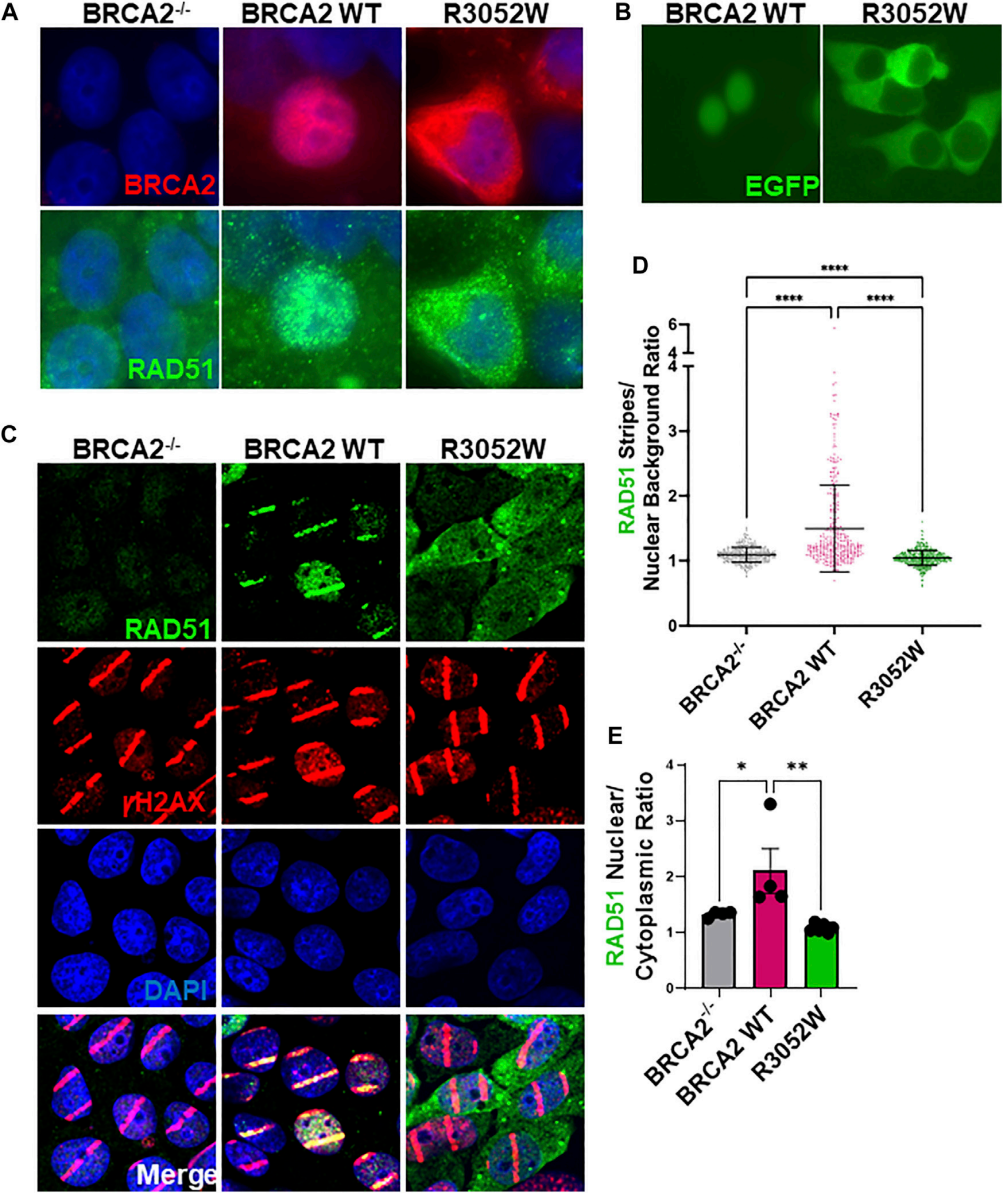
The R3052W protein localizes to the cytosol. **(A)** Immunofluorescent localization of BRCA2 in untreated BRCA2 knockout cells (BRCA2^−/−^), and stable cell lines expressing either BRCA2 WT or R3052W. Representative images of 2XMBP-BRCA2 (red, anti-MBP), RAD51 (green), and nuclei (blue). **(B)** Live images of BRCA2 knockout cells expressing either BRCA2 WT or R3052W fused to GFP at the N-terminus. **(C)** Representative immunofluorescence images of laser micro-irradiation experiments in BRCA2 knockout cells stably expressing BRCA2 WT or R3052W. DNA damage (stripes) are depicted in red (gammaH2AX), green (RAD51), and nuclei in blue (DAPI). **(D)** Quantification of RAD51 fluorescence intensity in damage areas (stripes) over the background in non-irradiated areas of respective laser micro-irradiated nuclei. Each data point represents a single analyzed nucleus, while the solid line is a mean value ± SD (Kruskal-Wallis test with Dunn’s multiple comparison post hoc test; **** *p* value < 0.0001). **(E)** Quantification of RAD51 intensity in the nuclear and cytoplasmic compartments. Each data point represents a single analyzed area (220.16 microns × 220.16 microns), while bars represent mean ± SD (one-way ANOVA with Holm-Šídák’s multiple comparisons post hoc test, * *p*-value<0.05; ** *p*-value<0.01).

In order to execute genomic integrity functions in both HDR of DNA DSBs and replication fork protection, it is essential that BRCA2 localizes to the nucleus (Spain et al., 1999;Jeyasekharan et al., 2013). To date, studies with BRCA2 fragments by the Venkitaraman group have shown that missense mutations in the DBD, including the R3052W mutation, are mislocalized to the cytosol due to impairment of DSS1 binding and/or active nuclear export (Jeyasekharan et al., 2013; Lee et al., 2021). However, these studies did not fully explore the cellular localization of all variants tested using the full-length BRCA2 protein nor did they address any changes in localization following DNA damage. We directly examined the localization of stably expressed WT BRCA2 and the R3052W mutant in our human BRCA2 knockout cell model by immunofluorescence under basal conditions (no exogenous DNA damage) **(** Figure 3A, quantification Supplementary Figure S2A,B **)**. To our surprise, and in agreement with studies using a BRCA2 fragment (Lee et al., 2021), the full-length R3052W protein localized to the cytosol **(** Figure 3A, upper right panel**)** whereas WT BRCA2 localized to the nucleus as expected **(** Figure 3A upper center panel**)** in most cells. Furthermore, transient transfection of WT BRCA2 and R3052W constructs fused at the N-terminus with GFP or mCherry in human 293T cells verified nuclear localization of the WT protein and cytoplasmic localization of R3052W utilizing live cell imaging (Figure 3B, Supplementary Figure S2C). As previously described by Lee et al., we confirmed that BRCA2 DBD + CTD localizes to the nucleus whereas R3052W DBD + CTD localizes to the cytosol (Supplementary Figure S2D). Finally, we found that the cellular localization of RAD51 correlated with the cellular compartment occupied by BRCA2 as most RAD51 signal was nuclear in cells expressing WT BRCA2 whereas the majority of RAD51 signal was present in the cytoplasm in R3052W cells (Figure 3A, lower panels, and quantification in Supplementary Figure S2B). By immunofluorescence, RAD51 appears as a diffuse signal in BRCA2 knockout cells (Figure 3A, lower left panel and quantification in Supplementary Figure S2B) likely due to lower expression levels (Magwood et al., 2013; Chatterjee et al., 2016) and loss of compartmentalization regulated by BRCA2. These results suggest that a large cellular pool of RAD51 is complexed with BRCA2 as proposed previously (Reuter et al., 2014).

We utilized laser microirradiation (LMI) to determine whether RAD51 could still be recruited to DNA damage and directed to a discrete nuclear location inside cells expressing the R3052W mutant. LMI allows for the creation of designated DNA damage sites within cell nuclei, often referred to as a “stripe”, and subsequent analysis of protein accumulation or modification. BRCA2^−/−^ cells stably expressing WT BRCA2 or R3052W were pre-treated with BrdU for 48 h and subsequently micro-irradiated with a 405 nm laser. Several localized DNA damage areas (stripes) were obtained as monitored by γH2AX Ser 139 phosphorylation (Figure 3C). At 30 min -post-micro-irradiation, prominent recruitment of RAD51 to damaged sites was observed in WT BRCA2 cells, whereas cells lacking BRCA2 displayed no RAD51 signal despite equal amounts of DNA damage as visualized by γH2AX stripe intensity (Figure 3C). Intriguingly, in BRCA2^−/−^ cells expressing R3052W, we observed diffuse nuclear/cytoplasmic staining of RAD51, similar to results under basal conditions (no exogenous DNA damage), with no discernible RAD51 signal localized to the γH2AX stripes (compare Figures 3A,C, RAD51 panels and quantification in Supplementary Figure S2B). We quantified intensities of protein recruitment to the laser-induced stripes using an ImageJ-based high-throughput tool in a minimum of 300 nuclei (Oeck et al., 2019). We normalized the fluorescence intensity of RAD51 protein staining present in each damaged area (stripe) to the overall background fluorescence in undamaged areas of the nucleus. Notably, the RAD51 stripe intensity was significantly higher in the WT BRCA2 cells than in BRCA2^−/−^ or R3052W expressing cells (Figure 3D). Additionally, we measured the overall mean fluorescence intensities of the RAD51 protein signal in nuclear and cytoplasmic compartments upon laser microirradiation. Strikingly, the ratio of nuclear to cytoplasmic RAD51 protein fluorescent intensity was significantly higher for WT BRCA2 cells than for BRCA2^−/−^ or R3052W expressing cells (Figure 3E). Overall, the results indicate that the R3052W mutation prevents significant accumulation of RAD51 in the nucleus and further impairs RAD51 recruitment to sites of DNA damage.

### R3052W Binds DSS1 and Remains Cytoplasmic Despite Nuclear Export Inhibition

**FIGURE 4:**
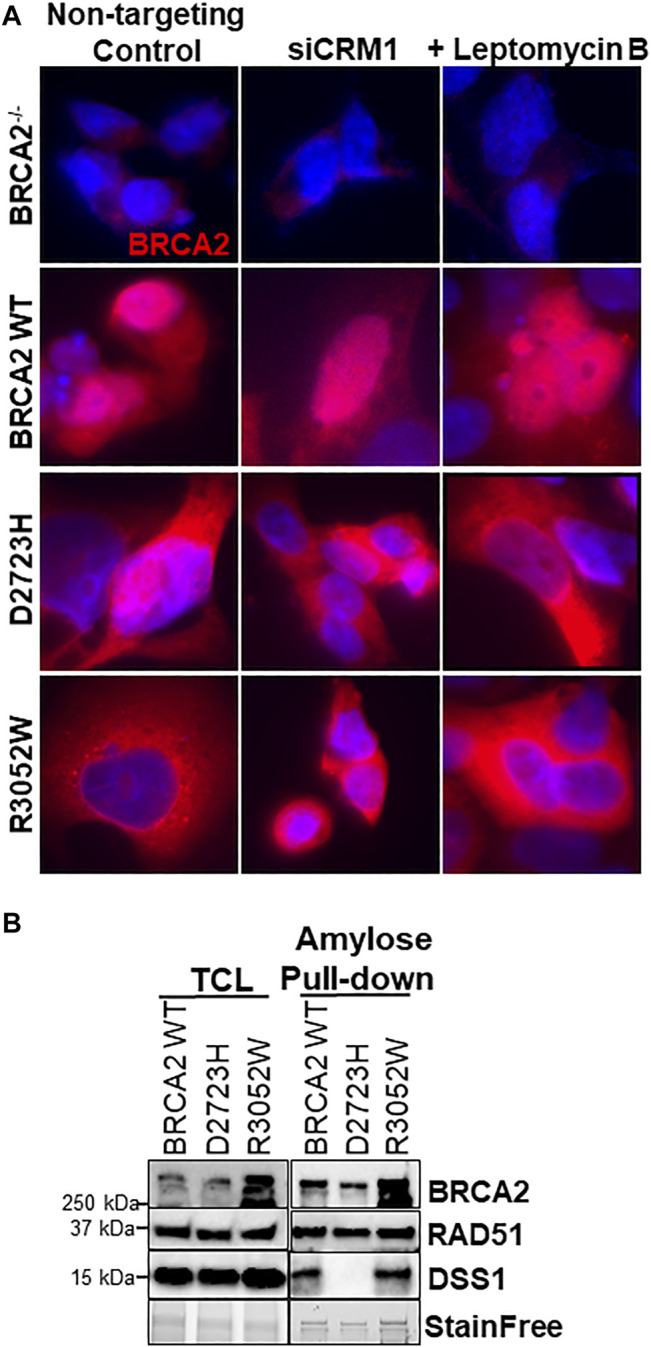
R3052W cytoplasmic localization is not altered by CRM1 depletion or leptomycin treatment and retains binding to DSS1. **(A)** Immunofluorescent localization of BRCA2 in untreated stable cell lines expressing WT, D2723H, or R3052W BRCA2 proteins upon RNA interference-mediated depletion of CRM1 or treatment with the nuclear export inhibitor leptomycin B. Representative images of BRCA2 (red, MBP antibody) and DAPI staining to visualize nuclei (blue). **(B)** Western blots of total cellular lysates (TCL) and amylose pulldowns from HEK 293T cells transiently transfected with 2XMBP-BRCA2 WT, D2723H, or R3052W co-transfected with HA-DSS1. Anti-MBP antibody was used for BRCA2 detection, Anti-RAD51 antibody was used for endogenous RAD51 detection and Anti-HA antibody was used for DSS1 detection.

**FIGURE 5:**
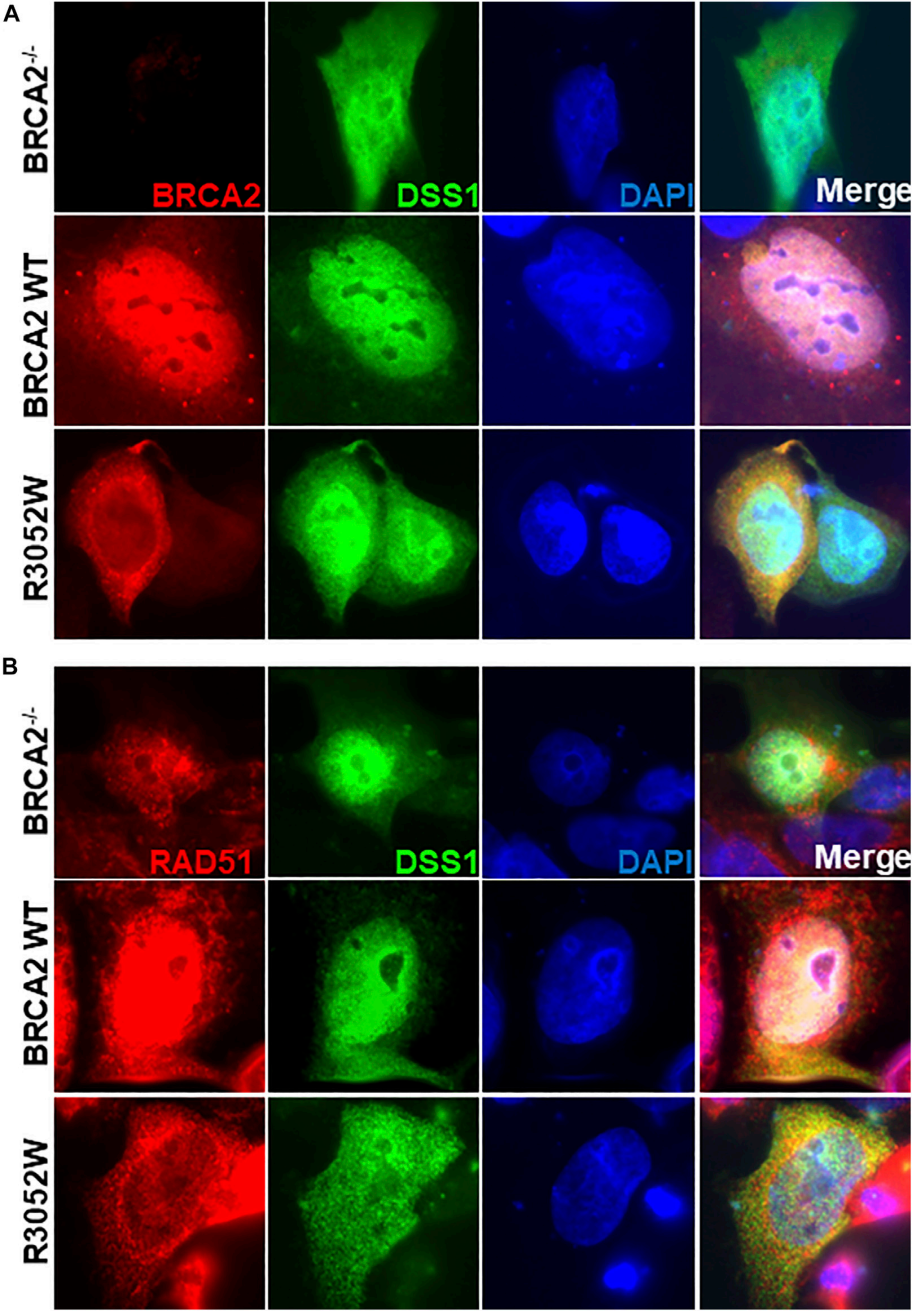
Ectopic expression of DSS1 does not alter BRCA2 WT, R3052W or RAD51 cellular localization. **(A)** Immunofluorescent localization of BRCA2 and DSS1 in untreated BRCA2 knockout cells stably expressing WT BRCA2 or the R3052W mutant concurrent with transient expression of HA-DSS1. Representative images of BRCA2 (red, MBP antibody), DSS1 (green, HA antibody) and DAPI staining to visualize nuclei (blue). **(B)** Immunofluorescent localization of RAD51 and DSS1 in untreated BRCA2 knockout cells stably expressing WT BRCA2 or the R3052W mutant concurrent with transient expression of HA-DSS1. Representative images of RAD51 (red, RAD51 antibody), DSS1 (green, HA antibody), and DAPI staining to visualize nuclei (blue).

A previous report interrogating the mislocalization of another BRCA2 missense mutation, D2723H, demonstrated that loss of DSS1 binding led to the exposure of a masked nuclear export signal sequence resulting in nuclear export to the cytoplasm (Jeyasekharan et al., 2013). The prior study utilized a fragment of BRCA2 (amino acids 2,432-3,418) containing the D2723H mutation, nonetheless, we confirmed that the full-length BRCA2 D2723H protein does indeed mislocalize to the cytoplasm (Figure 4A) and does not bind DSS1 (Figure 4B). However, in contrast to the prior study, we found that the full-length BRCA2 D2723H protein remained cytoplasmic despite CRM1 (exportin 1) depletion using RNA interference or by treatment with leptomycin B or Selinexor, two nuclear export inhibitors (Figure 4A, quantification in Supplementary Figures S2E, S3). To confirm the activity of leptomycin B in preventing nuclear export, we visualized nuclear retention of c-abl and Rev1.4 MP2K2 as positive controls (Supplementary Figures S3A,B). Likewise, the R3052W protein remained localized in the cytoplasm following the same treatments (Figure 4A, lower panel, and Supplementary Figures S3C,D). However, unlike D2723H, the R3052W protein retained binding to DSS1 (Figure 4B). We further over-expressed recombinant HA-DSS1 to determine if the R3052W protein could be re-directed to the nucleus, however, we failed to observe any significant movement (Figure 5A and quantification in Supplementary Figure S4). Interestingly, the cellular distribution of exogenously expressed HA-DSS1 appeared both nuclear and cytosolic (green panels in Figures 5A,B) in BRCA2^−/−^ cells (and in R3052W expressing cells) whereas a much higher nuclear intensity was observed in cells expressing nuclear WT BRCA2. Thus, WT BRCA2, but not R3052W, localizes with DSS1 in the nucleus. Moreover, RAD51 cellular localization correlated with BRCA2 independently of DSS1 (Figure 5B). In summary, the results confirm that the BRCA2 R3052W mutant protein is mislocalized to the cytoplasm, but unlike the D2723H variant, our results point towards a different mechanism than the loss of DSS1 binding leading to nuclear export. Further investigation will be required to reveal the mechanism underlying the cytoplasmic mislocalization of R3052W.

## Discussion

Our results confirm and extend previous reports demonstrating that arginine to tryptophan substitution at the highly conserved 3,052 residues, located in the DNA binding domain of BRCA2, alters critical HDR functions (Kuznetsov et al., 2008; Guidugli et al., 2013; Hart et al., 2019; Ikegami et al., 2020). We conclude that R3052W is a pathogenic mutation unable to perform HDR or rescue sensitivity to DNA damaging agents. Moreover, our findings of increased genomic instability (micronuclei formation) and sensitivity to MMC upon ectopic expression of R3052W in a background of wild-type endogenous BRCA2 suggest R3052W is a dominant negative allele. Dominant negative effects have been attributed to other missense variants in DNA repair proteins such as p53 and BRCA1 (Willis et al., 2004; Vaclova et al., 2016), re-affirming that loss-of-heterozygosity leading to loss-of-function is not the only path to increased cancer risk. Further analysis of the R3052W allele in a heterozygous state by knock-in at the endogenous locus should aid clarification of the dominant negative impact, however, gene targeting of endogenous BRCA2 is currently an extremely difficult technical challenge. Notably, the BRCA2 R3052W mutant protein maintains all RAD51 binding sites intact. R3052W mislocalization to the cytoplasm likely explains the dominant negative effect as the mutant protein could antagonize the wild-type nuclear BRCA2 by sequestering a portion of the cellular pool of RAD51 resulting in sub-optimal RAD51 recruitment to DSBs.

Despite the sequence identification and previous characterization of several potential NLS sites in BRCA2, it remains unclear if one or multiple NLS are necessary and sufficient for nuclear localization (Spain et al., 1999; Yano et al., 2000; Han et al., 2008; Jeyasekharan et al., 2013). BRCA2 interactions with PALB2 acting collaboratively to deliver RAD51 to sites of DNA damage sites may indirectly play a role in the nuclear retention of the BRCA2 protein (Xia et al., 2006). Previous studies suggested that mutations in (or near) the binding pocket of DSS1 could unmask a nuclear export sequence misdirecting BRCA2 to the cytosol through active nuclear transport (Jeyasekharan et al., 2013). During the course of our studies, Lee et al. observed that the R3052W mutation, in the context of a fragment comprising the BRCA2 DBD + CTD domain, was indeed localized to the cytosol (Lee et al., 2021). Our findings utilizing the full-length BRCA2 R3052W protein corroborate this result, however, discrepancies have arisen regarding the underlying molecular mechanism. Our results suggest R3052W mislocalization is independent of active nuclear export or DSS1 expression/binding. We directly demonstrate that full-length BRCA2 R3052W protein binds DSS1 and silencing of exportin 1 (CRM1) or inhibition of nuclear export by Leptomycin B or Selinexor treatments do not change the cytoplasmic location of mutant BRCA2 (Figure 4 and Supplementary Figure S3). Furthermore, ectopic expression of DSS1 does not alter the cellular location of the wild-type or mutant BRCA2 proteins but WT BRCA2 localizes with DSS1 in the nucleus (Figure 5).

We postulate that R3052W, and other pathogenic mutations in the BRCA2 DBD domain, are cytosolic due to either aggregation properties driven by protein misfolding or a breakdown in the recognition or regulation of the nuclear import machinery required to transport BRCA2 into the nucleus. Our analyses are ongoing and will hopefully shed light on the nature of the altered molecular mechanism. Immunohistochemical analyses capable of differentiating nuclear from cytoplasmic BRCA2 protein may present diagnostic opportunities to pathologically classify BRCA2 status in patient tumors.

As germline and tumor sequencing endeavors become incorporated into clinical cancer care, findings of missense variants, such as BRCA2 R3052W, with potentially uncertain functional consequences will be encountered more frequently (Thompson et al., 2001; Karchin et al., 2008; Guidugli et al., 2014). However, BRCA2 variants are present amongst healthy individuals and only a subset are causative of hereditary breast and ovarian cancer. The lack of classification standards and the rare incidence of individual variants can complicate the evaluation and diagnosis of patients. We advocate for the incorporation of functional assays into clinical practice to facilitate the correct classification of those BRCA2 missense variants where genetic linkage data and other traditional variant risk analyses are lacking. The ability to differentiate pathogenic from benign variants will enhance precision medicine efforts to stratify patients for increased cancer surveillance or targeted therapies such as PARP inhibitors.

## Data Availability

The raw data supporting the conclusions of this article will be made available by the authors, without undue reservation.
