# Supplementary material for: The Pathogenic R3052W BRCA2 Variant Disrupts Homology-Directed Repair by Failing to Localize to the Nucleus
Source: Front Genet. 2022 May 30;13:884210. doi: 10.3389/fgene.2022.884210 (PMC9197106; doi:10.3389/fgene.2022.884210)
Supplement: Supplementary file 1 [file DataSheet1.PDF]

Supplementary Materials & Methods

Sequence alignment and model R3052W

BRCA2 amino acid sequences from different organisms were obtained from *Uniprot Knowledge* database http://www.uniprot.org/ (Consortium et al 2011). Alignments were done with Bioedit http://www.mbio.ncsu.edu/bioedit/bioedit.html (Hall, 1999). The UniprotKB codes of the sequences used were*: Homo sapiens*, P5187; *Drosophila Melanogaster*, Q9W157; *Mus musculus*, P97929; *Felix Catus*, Q864S8; *Rattus norvegicus*, O35923; *Strongylocentrotus purpuratus*, A5A3F7; *Monodelphis domestica*, A4zz89; *Canis lupus familiaris*, Q8MKI9; *Xenopus tropicalis*, A4ZZ90. Swiss-Model Expasy server was used to model the change of an arginine to a tryptophan at position 3052 in the PDB structure 1IYJ (Yang et al., 2002;Waterhouse et al., 2018).

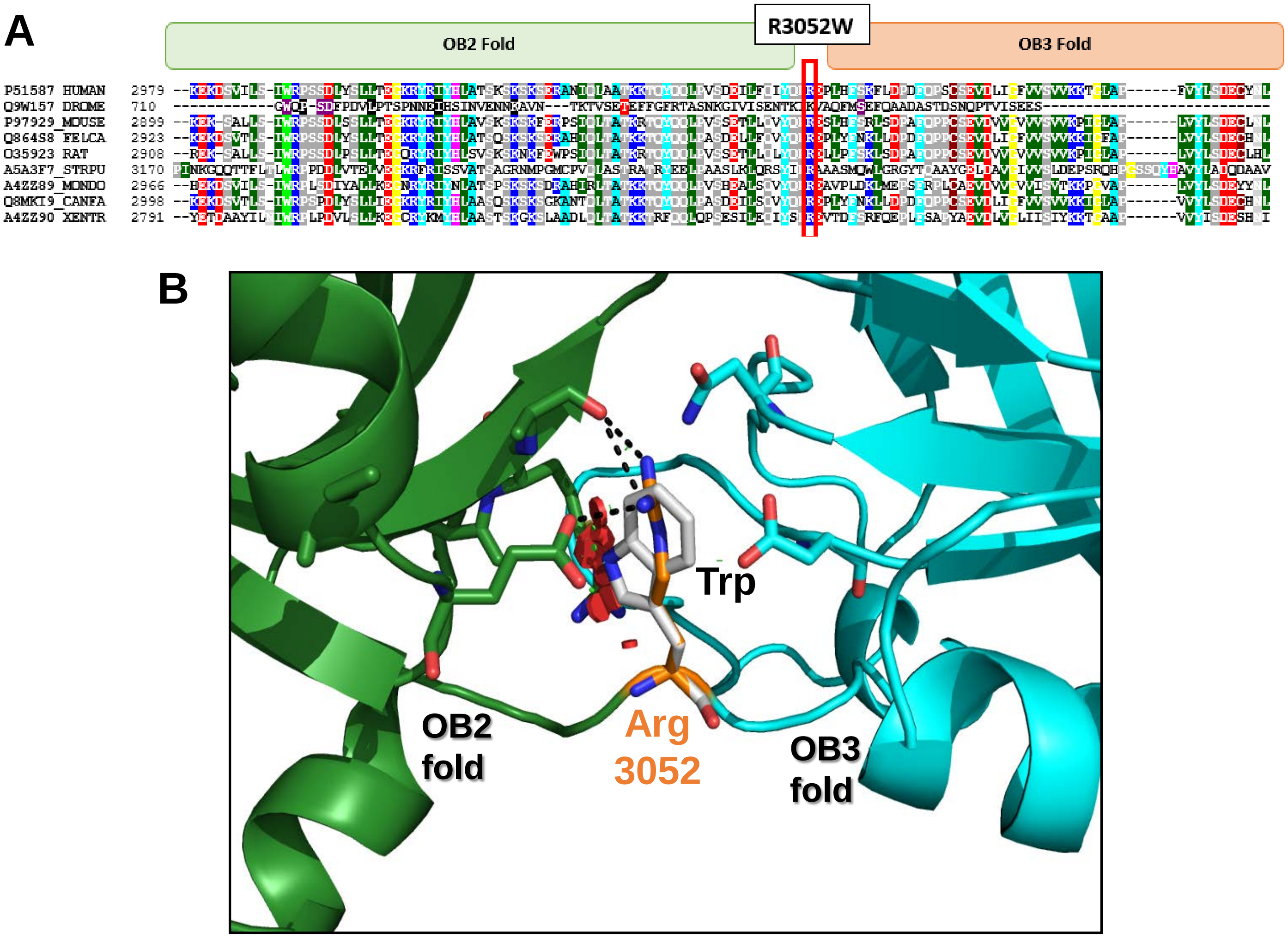

Supplementary Figure 1: BRCA2 residue R3052 is highly conserved amongst different species and substitution with tryptophan is predicted to disrupt interactions between the OB2-OB3 domains. A) Multiple Sequence alignment of BRCA2 amino acid sequences from different organisms flanking the R3052 residue. R3052 is indicated in a red box. BRCA2 amino acid sequences were obtained from Uniprot and ClustalX (70% threshold for shading) was used for the alignment. **B)** Structural model based on 1IYJ structure. The arginine to tryptophan substitution is displayed in grey. Clashes are indicated in red.

Supplementary Figure 1

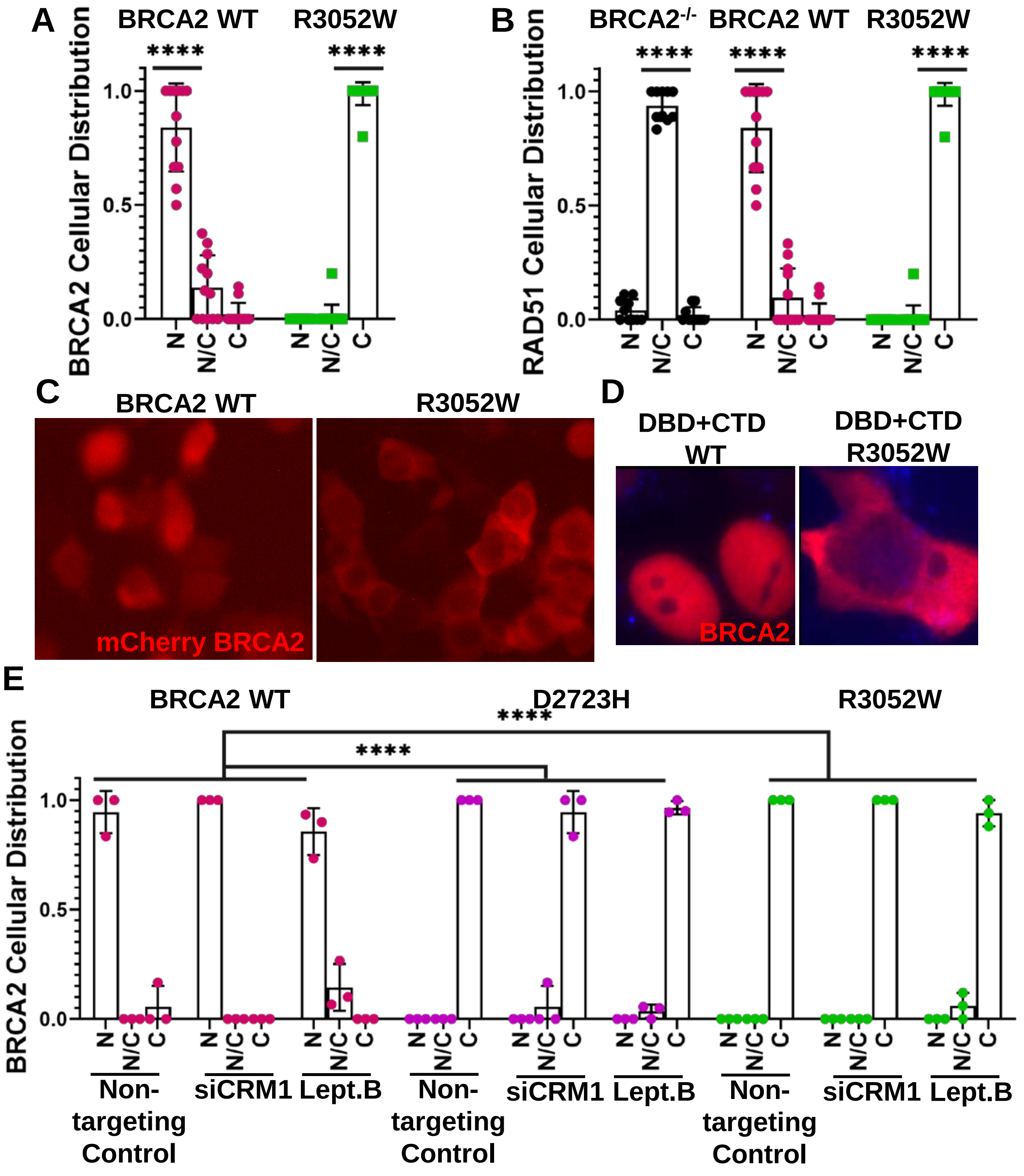

Supplementary Figure 2: R3052W is mislocalized to the cytosol. Quantification of **A)** BRCA2 and **B)** RAD51 localization in stable cell lines expressing either WT (pink) or R3052W (green) BRCA2 proteins. **C)** Live images of mCherry-BRCA2 WT or mCherry-R3052W transiently transfected in DLD-1 BRCA2 knockout cells. **D)** Localization by immunofluorescence of DBD+CTD WT and DBD+CTD R3052W. Representative images of 2XMBP-DBD+CTD (red, anti-MBP) and nuclei (blue). **E)** Quantification of BRCA2 localization in stable cell lines expressing WT (pink), D2723H (purple) or R3052W (green) BRCA2 proteins upon RNA-interference mediated depletion of CRM1 (siCRM1) or treatment with the nuclear export inhibitor leptomycin B (Lept. B). N (Nuclei), N/C (Nuclei/Cytosol), C (Cytosol). Bars represent mean ± SD. Statistical analysis: Multiple t-tests or two-way ANOVA to compare N and C in the different cell lines ****p value<0.0001.

Supplementary Figure 2

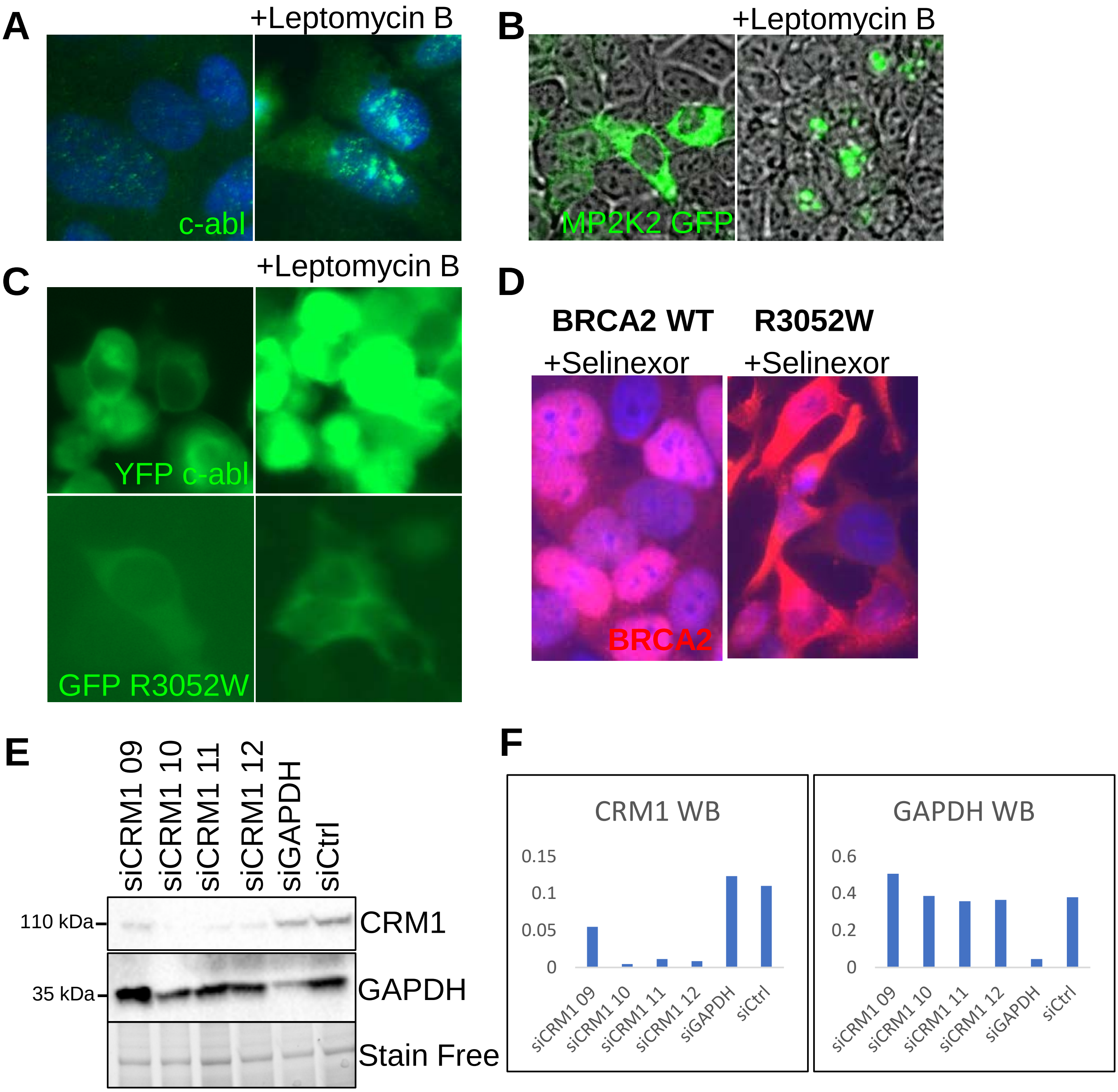

Supplementary Figure 3: Controls for nuclear export inhibitor leptomycin B and silencing of CRM1. A GFP tagged BRCA2 R3052W fusion protein does not localize to the nucleus upon leptomycin B treatment. A) Localization by immunofluorescence of c-abl with and without leptomycin B treatment (positive control). Representative images of c-abl (green, c-abl antibody) and DAPI staining to visualize nuclei (blue). **B)** Live images of MP2K2-GFP transiently transfected in DLD-1 BRCA2 knockout cells with and without Leptomycin B treatment as a positive control. **C)** Live images of YFP-c-abl (positive control) and GFP-R3052W transiently transfected in DLD-1 BRCA2 knockout cells with and without leptomycin B treatment. **D)** Immunofluorescent localization of BRCA2 in Selinexor treated cells stably expressing WT or R3052W BRCA2 proteins. **E)** Western blot of total cellular lysates from DLD-1 BRCA2 knockout cells silenced for 72 hours with CRM1 siRNA or GAPDH siRNA (control). **F)** Quantification of siRNA western blots. CRM1 and GAPDH antibodies were used for CRM1 and GAPDH detection, respectively. WB: Western Blot

Supplementary Figure 3

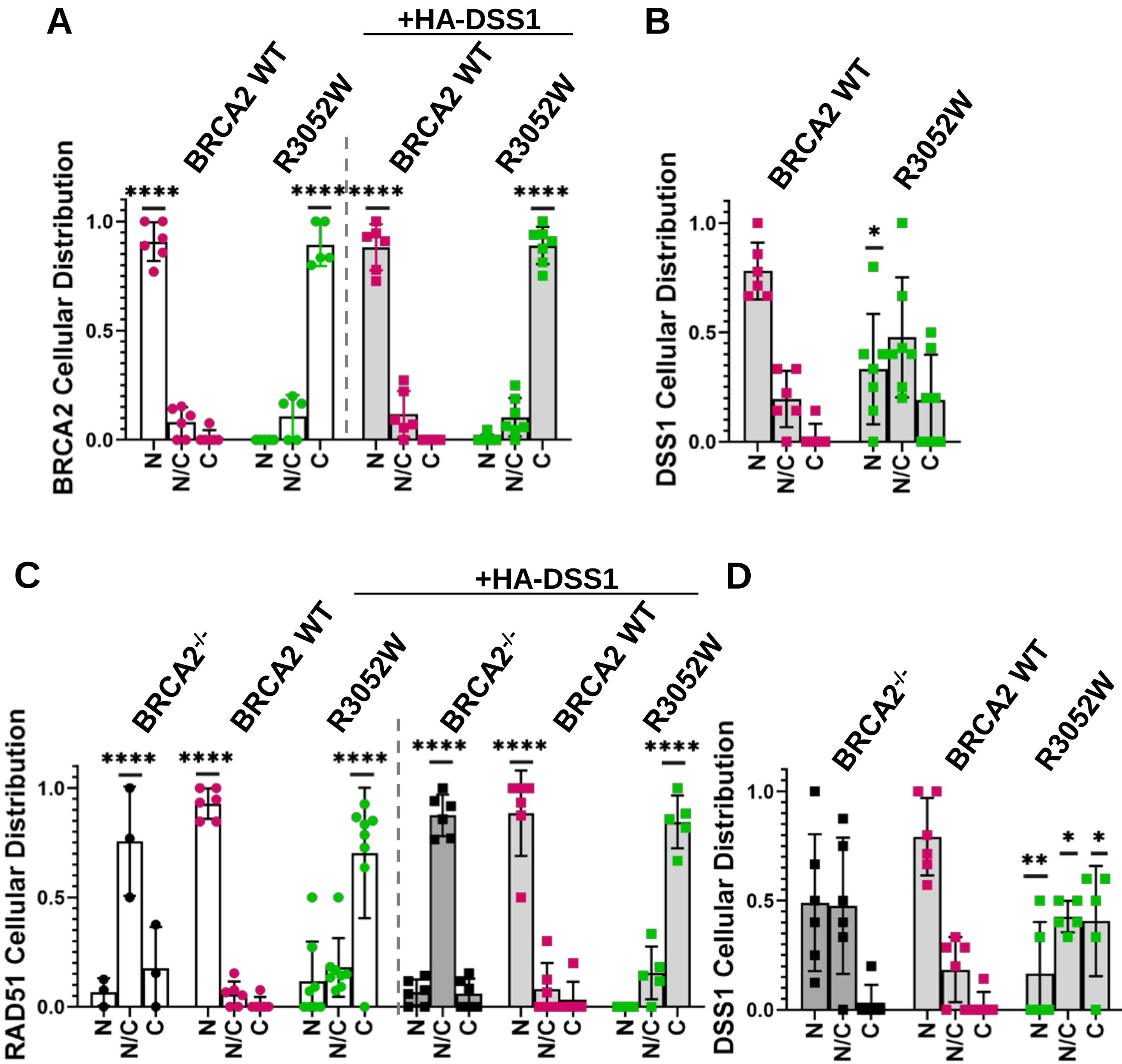

Supplementary Figure 4: Expression of DSS1 does not alter the localization of BRCA2. Quantification of **A)** BRCA2 and **C)** RAD51 localization in BRCA2$^{-/-}$ cells or stable cell lines expressing either WT (pink) or R3052W (green) BRCA2 proteins with (grey bars) and without (white bars) ectopic expression of DSS1. **B) and D)** Quantification of DSS1 localization in BRCA2$^{-/-}$ cells or stable cell lines expressing either WT (pink) or R3052W (green) BRCA2 proteins corresponding respectively to A) and C). N (Nuclei), N/C (Nuclei/Cytosol), C (Cytosol). Bars represent mean ± SD. Statistical analysis: Multiple t-tests or two-way ANOVA to compare N, N/C and C in the different cell lines *p value<0.01, ** p<0.001, **** p<0.00001.
